# Global proteomic analysis of plasma from mice infected with *Plasmodium berghei *ANKA using two dimensional gel electrophoresis and matrix assisted laser desorption ionization-time of flight mass spectrometry

**DOI:** 10.1186/1475-2875-10-205

**Published:** 2011-07-26

**Authors:** Evelyn N Gitau, Gilbert O Kokwaro, Charles RJC Newton, Stephen A Ward

**Affiliations:** 1 KEMRI-Wellcome Trust Collaborative Programme, Centre for Geographic Medicine Coast, Kilifi, Kenya; 2 Liverpool School of Tropical Medicine, Pembroke Place, Liverpool, UK; 3 Institute of Child Health, University College of London, London, UK; 4 Department of Pharmaceutics and Pharmacy Practice, School of Pharmacy, University of Nairobi, P. O. Box 19676-00202 (KNH), Nairobi, Kenya; 5 African Centre for Clinical Trials, P. O. Box 2288-00202 (KNH), Nairobi, Kenya; 6 Consortium for National Health Research, P.O. Box 29832-00202 (KNH), Nairobi, Kenya

## Abstract

**Background:**

A global proteomic strategy was used to identify proteins, which are differentially expressed in the murine model of severe malaria in the hope of facilitating future development of novel diagnostic, disease monitoring and treatment strategies.

**Methods:**

Mice (4-week-old CD1 male mice) were infected with *Plasmodium berghei *ANKA strain, and infection allowed to establish until a parasitaemia of 30% was attained. Total plasma and albumin depleted plasma samples from infected and control (non-infected) mice were separated by two-dimensional gel electrophoresis (2-DE). After staining, the gels were imaged and differential protein expression patterns were interrogated using image analysis software. Spots of interest were then digested using trypsin and the proteins identified using matrix-assisted laser desorption and ionization-time of flight (MALDI-TOF) mass spectrometry (MS) and peptide mass fingerprinting software.

**Results:**

Master gels of control and infected mice, and the corresponding albumin depleted fractions exhibited distinctly different 2D patterns comparing control and infected plasma, respectively. A wide range of proteins demonstrated altered expression including; acute inflammatory proteins, transporters, binding proteins, protease inhibitors, enzymes, cytokines, hormones, and channel/receptor-derived proteins.

**Conclusions:**

Malaria-infection in mice results in a wide perturbation of the host serum proteome involving a range of proteins and functions. Of particular interest is the increased secretion of anti-inflammatory and anti apoptotic proteins.

## Background

Malaria continues to affect millions of people in sub-Saharan Africa, where severe falciparum malaria is a major cause of childhood mortality. The malaria parasite infects 300-500 million people per year, causing over 1 million deaths [1]. In light of this, there is a need to better understand the biochemical changes associated with severe malaria as the definitive cause of death is often unknown.

Animal models of cerebral malaria have been developed to provide insight in to the pathogenesis of the disease although it is accepted that there are differences from the human condition. Cerebral malaria is induced in susceptible strains of mice by the ANKA strain of *Plasmodium berghei *[2]. These murine models of cerebral malaria have been used in the past to throw light on the pathogenesis of the human condition [3-6].

Proteome analysis is the direct measurement of all proteins in a system in terms of their presence and relative abundance at a specific point in time under defined conditions. Proteomics is regarded as complimentary technology to genome analysis. Proteins contain several dimensions that collectively indicate the actual rather than the potential functional state as indicated in mRNA analysis. Although the pattern of gene activity will be abnormal in a tissue with pathological lesions, there can be poor correlation between the level of activity of different genes and the abundance of their corresponding proteins within tissues [7]. Proteomic studies characterize the complex network of cell regulation at the protein level.

Here, the use of a global proteomic strategy to identify proteins differentially expressed in the murine model of cerebral malaria is reported. This work was conducted in order to identify opportunities for the development of novel diagnostic, disease monitoring techniques, and possible future treatments.

## Methods

### Mouse samples

Plasma was collected from 4-week-old CD1 male mice (n = 3) infected with the ANKA (PbA) strain of *P. berghei *originally obtained from the London School of Tropical Medicine and Hygiene (0.1 ml of a culture with 2% parasitaemia). A control set of plasma was collected from mice (n = 2) without infection. The samples were separated into two aliquots and albumin was depleted from one aliquot using the Montage Albumin kit (Millipore, USA). Proteomic analysis was carried out separately on each sample.

The use of mice in these experiments was undertaken in accordance with criteria outlined in a license granted under the Animals (Scientific Procedures) Act of 1986 and approved by the University of Liverpool Animal Ethics Committee.

### Two-dimensional electrophoresis (2-DE)

The first separation (first dimension) was carried out on a Multiphor II flat bed electrophoresis system (Pharmacia Biotech, Uppsala, Sweden). Ready-made strips Immobiline Drystrip gels (IPG) with a pH gradient 3-10 NL (non-linear) 13 cm long (Amersham Pharmacia Biotech, Bucks, UK) were used. The strips were passively rehydrated overnight at room temperature with a rehydration buffer (8 M Urea, 3% CHAPS, 0.5% IPG buffer 3-10, 10 mM DTT, and a trace amount of bromophenol blue), which contained 75 μg of plasma protein for preparative gels and 300 μg for analytical gels. Isoelectric focusing was carried out using multi-step conditions (1 h at 150 V, 1 h at 300 V, 1 h at 1500 V, 18 h at 3000V). Before the second dimension each strip was equilibrated by incubating for 15 minutes at room temperature in 10 ml of equilibration buffer (50 mM Tris-HCl, pH 8.8, 6 M urea, 30% w/v glycerol, 2% w/v SDS and a trace of Bromophenol Blue) with 1% w/v DTT. A second equilibrating step of 15 minutes was performed in 10 ml equilibration buffer containing 4%w/v iodoacetamide. The second dimension was performed on home made 12.5% homogenous vertical SDS-polyacrylamide gel slabs (gel plate size 180 × 160 mm and a gel thickness 1.5 mm) employing a Laemmli buffer system[8]. Electrophoresis was performed at 20°C under a constant current of 25 mA per gel using a Hoefer SE 600 series vertical slab gel electrophoresis unit (Amersham Pharmacia Biotech, Bucks, UK).

### Protein visualization and image analysis

Analytical gels were silver stained using a protocol described by Blum *et al *[9] with modifications described by Rabilloud *et al *[10]. Preparative gels were stained using colloidal Coomassie Brilliant Blue G-250 as previously described by Neuhoff *et al *[11]. The stained gels were scanned using a GS-710 Imaging Densitometer (BioRad, Hemel Hempstead, Hertfordshire UK). The gels were analysed using PDQuest^® ^software version 6.2.1 (Bio Rad, Hemel Hempstead, Hertfordshire UK) and Progenesis PG 220^® ^V2006 software (Nonlinear Dynamics, Newcastle upon Tyne, UK). PDQuest^® ^enabled the matching of identical spots in serial gels and normalization of gels to compensate for non-expression related variations in protein spot intensity. The software also enabled the tracking and reporting of all the protein patterns in the samples. By using the comparison tool for master gels, the software facilitated the comparison of differences between malaria infected and control groups. Semi-quantitative analysis of spots was performed using Progenesis PG220^®^.

### Tryptic in-gel digestion

Eppendorf tubes and all utensils were cleaned with 50% v/v acetonitrile/0.1% v/v TFA solution and dried. The protein spots of interest identified on preparative gels were excized and transferred to the Eppendorf tubes. The chopped gel pieces were washed in 50% acetonitrile/25 mM ammonium bicarbonate, pH 7.8, and dried in a vacuum concentrator. Then 4-10 μl digestion buffer (10 μg/ml modified sequencing grade trypsin (Promega) in 25 mM ammonium bicarbonate) was added to the dried gel pieces and incubated overnight at 37°C. Resulting peptides were extracted by addition of 4 μl water followed by 7 μl 30% acetonitirile/0.1% TFA followed by vortexing and brief centrifugation. The supernatant was transferred to a clean tube and vacuum concentrated to approximately 5 μl.

### Mass spectrometric identification of proteins

The concentrated sample (0.5 μl) was directly applied onto the sample target plate with equal amounts of matrix (10 mg/ml α-cyano-4-hydroxycinnamic acid (HCCA; Aldrich) in 50% acetonitrile/0.1% TFA). Mass spectra were obtained using MALDI-MS (Shimadzu CFR Plus, Manchester UK) in positive ion reflectron mode at an accelerating voltage of 20 kV. The spectra were externally calibrated using a peptide mixture (Sigma, St Louis, MO) with masses 757.39 (Bradykinin), 1046.54 (Angiotensin II) and 2465.19 (ACTH). Spectra obtained were used to search through the NCBI nr database using the MASCOT Peptide Mass Fingerprinting software [12] (with a tolerance of ~ +/- 0.1 D and one missed cleavage site.

## Results

### Protein separation by 2-DE

Plasma samples from infected mice together with control samples were applied to 2-DE and proteins visualized by silver stain for analytical gels and Coomassie blue stain for preparative gels. Representative gel maps for plasma from infected mice, and control mice before and after albumin depletion were created using PDQuest^®^. The gel maps are synthetic gels prepared by analysing four replicate silver stained gels of each sample. Representative gel master gels of controls, infected, albumin depleted control and infected had 752, 639, 921, and 610 spots respectively. Individual variation was calculated using PDQuest and extent of correlation of protein spots between the replicate gels was reported as correlation coefficients. For all gel maps, correlation between the individual gels was greater than 0.772. A coefficient of 1.00 indicates that two gels are perfectly similar, while a low coefficient (e.g. 0.40) indicates the two gels are not very similar. There were protein spots that were differentially expressed on the gel of plasma from infected mice and some proteins were completely absent. Removing albumin improved the quality of gels and helped identify some additional spots of interest.

### Mass spectrometry and protein identification

**Figure 1:**
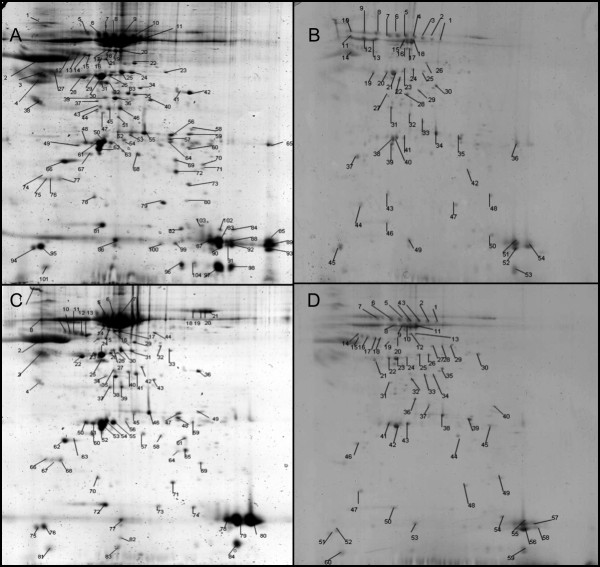
**Coomassie stained 2-D PAGE gel of plasma from mice after loading with 300 μg of sample protein**. Plasma from infected mice (A), albumin depleted plasma from infected mice (B), plasma from control mice (C) and albumin depleted plasma from control mice (D).

When stained with Coomassie between 60 and 104 spots per gel were visualized. Figure 1 shows the spots excized from the Coomassie stained gels. These spots were digested and prepared for MALDI analysis. It was possible to definitively identify 51, 63, 30 and 18 proteins from the infected, control, albumin depleted infected and albumin depleted control plasma samples respectively. Tables 1 and 2 give a list of these uniquely expressed proteins.

**Table 1:** List of proteins identified from the Coomassie stained gels of plasma from infected mice (Figure 2)

**Spot No**.	Description	Accession	Score	Matched Peptides	Sequence coverage(%)
1	serine (or cysteine) proteinase inhibitor, clade A, member 1a; serine protease [*Mus Musculus*]	15029662	83	8	26

12, 14	similar to macrophage galactose-type C-type lectin 2 [*Mus Musculus*]	38089618	78, 68	7, 6	21, 20

36	similar to spectrin alpha chain, brain (spectrin, non-erythrid alpha chain)	38074605	71	10	

42	60S ribosomal protein L35a	3914537	70	5	31

48, 50, 56, 72, 94, 95	apolipoprotein A-1 precursor - mouse	109571	137, 102,87,72,64,65	11, 9, 8, 7, 7, 6	34, 29, 27, 23, 19, 19

48, 50, 56, 67, 72, 94, 95	unamed protein product [*Mus Musculus*]	26345182	136, 102, 87, 76, 72, 64, 65	11, 9, 8, 8, 7, 7, 6	34, 29, 27, 28, 23, 19, 19

48, 50, 56, 94, 95	apolipoprotein A-1 [*Mus Musculus*]	2145135	119, 86, 72, 64, 65	10, 8, 7, 7, 6	29, 24, 22, 19, 19

48, 94	apolipoprotein A1 homolog [mus sp.]	1245804	69, 66	5, 7	53, 53

55	unamed protein product [*Mus Musculus*]	26341396	80	9	20

55	albumin 1; serum albumin variant [*Mus Musculus*]	33859506	78	9	19

55	serum albumin precursor	5915682	78	9	19

65	similar to 60S Ribsomal protein L29 (P23) [*Rattus Norvegicus*]	34866986	67	6	28

66	RNA binding motif protein, X chromosome [*Mus Musculus*]	6755296	72	8	16

66, 103	heterogenous nuclear ribonucleoprotein G [*Mus Musculus*]	5579009	68, 67	7, 7	20, 15

66	unamed protein product [*Mus Musculus*]	26339834	66	9	10

80	similar to 60S Ribsomal protein L29 (P23) [*Mus Musculus*]	38089196	78	7	25

80	similar to 60S Ribsomal protein L29 (P23) [*Rattus Norvegicus*]	34881340	71	7	30

81	unamed protein product [*Mus Musculus*]	26335691	69	7	25

81	heparan sulfate 6-0-sulfotransferase 1 [*Mus Musculus*]	20845347	64	6	28

82	similar to HRPAP20 short form [*Rattus Norvegicus*]	34867098	65	6	20

99	similar to Rp17a protein [*Rattus Norvegicus*]	34868324	86	9	16

The MALDI spectra were searched against the NCBInr database using the Mascot^® ^search algorithm. Proteins with a significant score as defined by Mascot^® ^were included in the list.

**Table 2:** List of proteins identified from the Coomassie stained gels of plasma from control mice

**Spot No**.	Description	Accession	Score	Matched Peptides	Sequence coverage (%)
7, 23, 24, 46, 70, 71	albumin 1; serum albumin variant [*Mus Musculus*]	33859506	104, 104, 92, 136, 77, 76	11, 11, 11, 14, 9, 9	22, 22, 25, 26, 18, 17

22	apolipoprotein A-IV [*M. musculus*]	29477189	80	8	26

28	hypothetical protein XP_125606 [*Mus Musculus*]	20858591	65	7	22

30	lectin, galactose binding, soluble 7 [*Mus Musclus*]	31543120	66	6	36

35	similar to 60S ribosomal protein L7a (Sufeit locus protein 3) PLA-X polypeptide	34869618	64	6	29

40	polyadenlate-binding protein 4 (PABP 4) [*Rattus Novegicus*]	27690704	81	11	16

44	hypothetical protein XP_218509 [*Rattus Norvegicus*]	34855811	72	9	9

46	unamed product [*Mus Musculus*]	2614396	140	14	26

50, 53, 54, 55, 61, 62, 64, 65	unamed product [*Mus Musculus*]	26345182	64, 123, 139, 92, 153, 118, 63, 86	7, 11, 12, 9, 12, 10, 6, 8	24, 35, 38, 29, 31, 35, 18, 29

51	Non0/p54nrb homolog [*Rattus Norvegicus*]	2674209	71	6	23

51	stress-induced phosphoprotein 1 [*Mus Musculus*]	13277819	69	9	9

52	cytochrome c oxidase subunit VIIc [*Mus Musculus*]	6680991	70	5	36

53, 54, 55, 61, 62, 64, 65	apolipoprotein A-1 [*Mus Musculus*]	6753096	123, 139, 68, 153, 118, 63, 86	11, 12, 9, 12, 10, 6, 8	35, 38, 29, 31, 30, 18, 29

67, 68	major urinary protein [mice]	1839508	85, 163	7, 11	36, 42

67	similar To RIKEN Cdna 1700001e04 [*Mus Musculus*]	38076876	62	6	21

70	albumin [*Mus Musculus*]	26986064	80	7	38

71	60S Ribosoml protein L4 (L1)	3914699	78	6	39

72	transthyretin [*Mus Musculus*]	7305599	97	7	20

72	unamed product [*Mus Musculus*]	12852317	64	5	34

75	similar to hypothetical protein FLJ25333 [*Mus Musculus*]	38075385	68	7	17

75	RNA binding motif protein, X chromosome [*Mus Musculus*]	6755296	64	7	13

77, 78, 79	Hemoglobin beta subnit 1 [*Mus Musculus*]	31982300	72, 74, 93	6, 6, 7	41, 41, 61

89	heterogenous nuclear ribonucleoprotein G [*Mus Musculus*]	5579009	67	7	18

In accordance with the cut-off score recommended by Mascot^® ^for *Rodents *a protein score of 63 was considered to be significant. Removing albumin improved the quality of gels and helped identify additional proteins. Semi-quantitative differential analysis was done on silver stained replicate gels using Progenesis PG 220^® ^2DE software. Spots were given a match number if 3 out of 4 replicates matched perfectly. Spots matched to each representative gel were then analysed for any changes in intensity.

### Functional cataloguing of proteins

**Figure 2:**
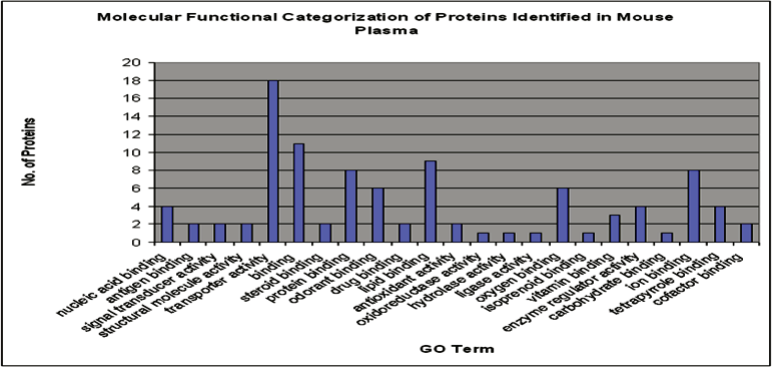
**Functional Categorization of 106 proteins identified based on gene ontology classification for Molecular Function**. The Gene Ontology (GO) numbers were derived from Protein Information Resource (PIR) -http://pir.georgetown.edu/ Batch retrieval tool. This tool converted the GI NCBInr accession numbers to GO numbers and then used GO slim to categorize the proteins. Another 47 identified proteins were unmatched to GO and not included in this graph.

**Figure 3:**
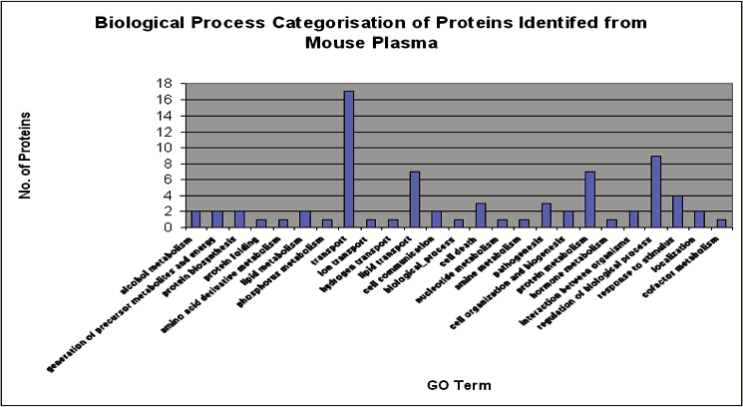
**Functional Categorization of 106 proteins identified based on gene ontology classification for Biological Process**. The Gene Ontology (GO) numbers were derived from Protein Information Resource (PIR) -http://pir.georgetown.edu/ Batch retrieval tool. This tool converted the GI NCBInr accession numbers to GO numbers and then used GO slim to categorize the proteins. Another 47 identified proteins were unmatched to GO and not included in this graph.

Protein functions were identified using the Protein Information Resource (PIR) id-mapping tool and catalogued according to their gene ontology (GO) number. The software enabled us to identify the molecular functions, cellular components and biological processes of the proteins. Figures 2 and 3 show a graphic breakdown of identified proteins according to their GO functional categories.

## Discussion

Animal models have been developed to provide insights into the pathogenesis and neurological complications of malaria. Mouse models have been studied most extensively to examine the function of the blood brain barrier in malaria. Pathology of the *Plasmodium berghei *ANKA mouse model used in this study develops neurological signs, such as ataxia, hemiplegia and coma [13], clinical features which are seen in human CM. However, in contrast to human CM, there is little evidence of sequestration of parasitized red blood cells (PRBCs) in the brain [14], but rather monocytes and non-PRBCSs predominate [5]. However, recent studies have found PRBCs in the cerebrum and cerebellum of infected mice [15].

Although the pathogenesis of the neurological complications of murine malaria appears to be more inflammatory [14,15] than in human malaria, changes in protein components of mouse plasma may provided insights into these complications.

Of the 157 proteins identified 54 were uniquely expressed in the plasma from infected mice. Most of the differentially or uniquely expressed proteins in plasma are acute phase proteins that are widely thought to indicate generalized inflammation and apoptosis. Other proteins identified suggest that the parasite may cause the host to express proteins that support cell invasion and reduce apoptosis, which would lead to better survival of infected red blood cells.

One of the unique proteins identified was similar to spectrin non-erythroid alpha chain brain protein (spot 36 infected gel map). This protein has recently been shown to correlate with severity of CM in Gabonese children [16]. Another of the proteins identified as unique to plasma from infected mice was related to macrophage galcotose-type C-type lectin 2 (MGL2) (Spot 12 and 14). MGL2 is induced in diverse populations of activated macrophages, including peritoneal macrophages during infection with the protozoan, *Trypanosoma brucei brucei *or the helminth, *Taenia crassiceps*; and alveolar macrophages in a mouse model of allergic asthma [17]. Raes *et al *[17] also demonstrated that *in vitro*, interleukin-4 (IL-4) and IL-13 up-regulate MGL2 expression and that *in vivo*, induction of MGL2 is dependent on IL-4 receptor signaling. Moreover, expression of MGL on human monocytes is also up-regulated by IL-4. The study concluded that macrophage galactose-type C-type lectins represent novel surface markers for murine and human activated macrophages and could be important markers of immune response in murine malaria.

Another of the proteins identified as unique to plasma drawn from infected mice is Baculoviral Inhibitor of Apoptosis (IAP) repeat-containing protein 3, which is an apoptotic suppressor. This protein interacts with tumour necrosis factor (TNF) receptor associated factors 1 and 2 to form an heteromeric complex, which is then recruited to the TNF receptor 2 [18]. The presence of this protein could be protective, trying to abrograte apoptosis caused by a massive local release of TNF in both murine liver cells during malarial infection [19,20] and in endothelial cells from human cerebral malaria [21,22]. Moreover the identification of a protein similar to hormone-regulated proliferation-associated protein 20 (HRPAP20) (spot 82 infected) suggests that in murine malaria suppression of apoptosis plays a key role in disease progression.

Two proteins associated with mediation of anti -inflammation, Apolipoprotein A1 (spot 42 infected depleted) and heparan sulfate transferase 1 (spot 99 infected) were also differentially expressed strengthening the idea that the mouse model is predominantly an inflammatory disease.

There were 49 proteins expressed in the control gels that were absent from malaria infected samples. These included transthyretin (spot 72 control gel), which sometimes acts as a retinol carrier through an association with retinol binding protein [23]. It is normally found at high concentrations in plasma and the fact that it was not detected in the plasma from infected mice would suggest that the levels in rodent malaria are too low to detect or some protein modification had occurred. Transthyretin is an acute phase protein that decreases during an acute phase response [24]. Together with other acute phase proteins such as transferrin and albumin, these proteins decrease during an acute phase response and have no apparent immune function [24]. Their main role is to transport nutrients therefore; their reduction during infection and inflammation may lower the concentration of specific nutrients. To support this hypothesis it has been shown that the serum concentration of retinol, the alcohol form of vitamin A, decreases during malarial infections. This reduction has been characterized as a direct consequence of the inflammatory response to *Plasmodium *infections [25,26].

Previously, several proteins have been associated with a reduction in retinol [27-29]. The proteomic results here suggest that transthyretin may be a useful predictor of plasma retinol during malarial infection. This information would be useful because the inclusion of a measure of the acute phase response would help interpret plasma retinol concentrations during malarial infection [25,27].

## Conclusion

This study goes some way to validate the technique of proteomics for characterising proteins differentially expressed during disease in this case with the *P. berghei *ANKA mouse malaria model. The results here suggest that apoptosis and inflammation play a major role in disease progression and also suggest that qualitative analysis of markers of activated macrophages could help elucidate their role in controlling murine malaria as well as confirming the relevance of macrophages in developing host immunity to this infection. As a whole, the study provides a "proof of concept" on use of plasma proteomics to help understand host response to the malaria parasite.

## Competing interests

The authors declare that they have no competing interests.

## Authors' contributions

EG carried out the proteomic experiments and drafted the paper. CJRC, SAW and GK participated in the design of the study and helped draft the manuscript. All authors read and approved the final manuscript.
